# What Are the Main Drivers of the Bitcoin Price? Evidence from Wavelet Coherence Analysis

**DOI:** 10.1371/journal.pone.0123923

**Published:** 2015-04-15

**Authors:** Ladislav Kristoufek

**Affiliations:** 1 Warwick Business School, University of Warwick, Coventry, West Midlands, CV4 7AL, United Kingdom, EU; 2 Institute of Information Theory and Automation, Academy of Sciences of the Czech Republic, Pod Vodarenskou vezi 4, Prague 8, 182 08, Czech Republic, EU; 3 Institute of Economic Studies, Charles University, Opletalova 26, 110 00, Prague, Czech Republic, EU; Universita' del Piemonte Orientale, ITALY

## Abstract

The Bitcoin has emerged as a fascinating phenomenon in the Financial markets. Without any central authority issuing the currency, the Bitcoin has been associated with controversy ever since its popularity, accompanied by increased public interest, reached high levels. Here, we contribute to the discussion by examining the potential drivers of Bitcoin prices, ranging from fundamental sources to speculative and technical ones, and we further study the potential influence of the Chinese market. The evolution of relationships is examined in both time and frequency domains utilizing the continuous wavelets framework, so that we not only comment on the development of the interconnections in time but also distinguish between short-term and long-term connections. We find that the Bitcoin forms a unique asset possessing properties of both a standard financial asset and a speculative one.

## Introduction

The Bitcoin [1] is a potential alternative currency to the standard fiat currencies (e.g., US dollar, the Euro, Japanese Yen) with various advantages such as low or no fees, a controlled and known algorithm for currency creation, and an informational transparency for all transactions. The Bitcoin’s success has ignited an exposition of new alternative crypto-currencies, usually labelled as “Altcoins”; however, none of these have been able to jeopardize the Bitcoin’s dominant role in the field. Of course, where there is an upside, there is often a downside as well. Simultaneously with its increasing popularity and public attention, the Bitcoin system has been labelled as an environment for organized crime and money laundering, and it has been a target of repeated hacker attacks that have caused major losses to some bitcoin owners [2, 3]. However, it should be noted that all of these issues can be a concern for standard cash currencies as well.

Though the Bitcoin has been frequently discussed on various financial blogs and even mainstream financial media, the research community is still primarily focused on the currency’s technical, safety and legal issues [2–7], but discussion about the economic and financial aspects remains relatively sparse. Bornholdt & Sneppen [8] construct a model with voter-like dynamics and show that the Bitcoin holds no special advantages over other crypto-currencies and might be replaced by a competing crypto-currency. Kondor *et al*. [9] study the Bitcoin network in a standard complex networks framework and show that the network characteristics of the Bitcoin evolve in time and that these are due to bitcoins increasing acceptance as a means of payment. Further, they show that the wealth in bitcoins is accumulating in time and that such accumulation is tightly related to the ability to attract new connections in the network. Garcia *et al*. [10] study Bitcoin bubbles using digital behavioral traces of investors in their social media use, search queries and user base. They find positive feedback loops for social media use and the user base. In our previous study [11], we focus on a speculative part of the Bitcoin value as measured by the search queries on Google and searched words on Wikipedia, showing that both the bubble and bust cycles of Bitcoin prices can be at least partially explained by interest in the currency. Following that study, the Bitcoin attracted even more attention when its exchange rate with the US dollar breached the $1000 level (with a maximum of $1242 per bitcoin at the Mt. Gox market, creating an absurd potential profit of more than 9000% for a buy-and-hold strategy in less than 11 months) in late November and early December 2013. After the subsequent corrections, the value of the Bitcoin has stabilized between $900 and $1000 per bitcoin at a break of years 2013 and 2014. However, a huge strike to the Bitcoin’s credibility and reputation came with the insolvency of the Mt. Gox exchange, historically the most prominent of the Bitcoin markets, after which the Bitcoin price started a slow stable decreasing trend with rather low volatility. At the end of the analyzed period (April 2014), a bitcoin traded between $400 and $500.

Here, we address the price of the Bitcoin currency, taking a wider perspective. We focus on various possible sources of price movements, ranging from fundamental sources to speculative and technical sources, and we examine how the interconnections behave in time but also at different scales (frequencies). To do so, we utilize continuous wavelet analysis, specifically wavelet coherence, which can localize correlations between series and evolution in time and across scales. It must be stressed that both time and frequency are important for Bitcoin price dynamics because the currency has undergone a wild evolution in recent years, and it would thus be naive to believe that the driving forces of the prices have remained unchanged during its existence. In addition, the frequency domain viewpoint provides an opportunity to distinguish between short- and long-term correlations. We show that the time and frequency characteristics of the dynamics are indeed both worth investigating, and various interesting relationships are uncovered.

## Methods

Before turning to the results of our analysis, we provide a detailed description of the utilized wavelets methodology. In this section, we also provide a descriptive list of the data sources, which are crucial for the whole analysis, as he data availability of Bitcoin is unique in comparison with other financial assets.

### Wavelets

A wavelet *ψ*(*t*) is a complex-valued square integrable function generated by functions of the form

$$\begin{array}{c} \psi_{u,s}\left(t\right)=\frac{\psi (\frac{t-u}{s})}{\sqrt{s}} \end{array}$$ (1)

with scale *s* and location *u* at time *t*. Given the admissibility condition [12], any time series can be reconstructed back from its wavelet transform. A wavelet has a zero mean and is standardly normalized so that $\int_{-\infty }^{+\infty }\psi (t)dt=0$ and $\int_{-\infty }^{+\infty }|\psi {|}^{2}(t)dt=1$. A continuous wavelet transform *W*_*x*_(*u*, *s*) is obtained via the projection of a wavelet *ψ*(.) on the examined series *x*(*t*) so that

$$\begin{array}{c} W_{x}\left(u,s\right)=\int_{-\infty }^{+\infty }\frac{x\left(t\right)\psi^{*}(\frac{t-u}{s})dt}{\sqrt{s}} \end{array}$$ (2)

where ψ*(.) is a complex conjugate of *ψ*(.). The original series can be reconstructed from the continuous wavelet transforms for given frequencies so that there is no information loss [13, 14]. From a wide range of complex-valued wavelets that allow for a multivariate analysis, we opt for the Morlet wavelet, which provides a good balance between time and frequency localization [14, 15].

The continuous wavelet framework can be generalized for a bivariate case to study the relationship between two series in time and across scales. A continuous wavelet transform is then generalized into a cross wavelet transform as

$$\begin{array}{c} W_{xy}\left(u,s\right)=W_{x}\left(u,s\right)W_{y}^{*}\left(u,s\right) \end{array}$$ (3)

where *W*_*x*_(*u*, *s*) and *W*_*y*_(*u*, *s*) are continuous wavelet transforms of series *x*(*t*) and *y*(*t*), respectively [16]. As the cross wavelet transform is in general complex, the cross wavelet power ∣*W*_*xy*_(*u*, *s*)∣ is usually used as a measure of co-movement between the two series. The cross wavelet power uncovers regions in the time-frequency space where the series have common high power, and it can be thus understood as a covariance localized in the time-frequency space. However, as for the standard covariance, the explanation power of ∣*W*_*xy*_(*u*, *s*)∣ is limited because it is not bounded.

To address this weakness, the wavelet coherence is introduced as

$$\begin{array}{c} R_{xy}^{2}\left(u,s\right)=\frac{{\left|S(\frac{1}{s}W_{xy}\left(u,s\right))\right|}^{2}}{S(\frac{1}{s}{\left|W_{x}\left(u,s\right)\right|}^{2})S(\frac{1}{s}{\left|W_{y}\left(u,s\right)\right|}^{2})}, \end{array}$$ (4)

where *S* is a smoothing operator [14, 17]. The squared wavelet coherence ranges between 0 and 1, and it can be interpreted as a squared correlation localized in time and frequency. Due to the above mentioned complexity of the used wavelets and in turn the use of the squared coherence rather than coherence itself, information about the direction of the relationship is lost. For this purpose, a phase difference is introduced as

$$\begin{array}{c} \varphi_{xy}\left(u,s\right)=\tan^{-1}(\frac{ℑ[S(\frac{1}{s}W_{xy}\left(u,s\right))]}{ℜ[S(\frac{1}{s}W_{xy}\left(u,s\right))]}), \end{array}$$ (5)

where ℑ and ℜ represent an imaginary and a real part operator, respectively. Graphically, the phase difference is represented by an arrow. If the arrow points to the right (left), the series are positively (negatively) correlated, i.e., they are in the in-phase or the anti-phase, respectively, and if the arrow points down (up), the first series leads the other by $\frac{\pi }{2}$ (vice versa). The relationship is usually a combination of the two, i.e., if the arrow points to the northeast, the series are positively correlated and the second series leads the first. Note that the interpretation of phase relationships is partially dependent on specific expectations about the relationship because a leading relationship in the in-phase can easily be a lagging relationship in the anti-phase. Please refer to Ref. [14] for a detailed description.

Recently, the partial wavelet coherence has been proposed to control for the common effects of two variables on the third [18, 19], and it is defined as

$$\begin{array}{c} RP_{y,x_{1},x_{2}}^{2}=\frac{{\left|R_{yx_{1}}-R_{yx_{2}}R_{yx_{1}}^{*}\right|}^{2}}{{\left(1-R_{yx_{2}}^{2}\right)}^{2}\;{\left(1-R_{x_{2}x_{1}}^{2}\right)}^{2}}. \end{array}$$ (6)

The partial wavelet coherence ranges between 0 and 1, and it can be understood as the squared partial correlation between series *y*(*t*) and *x*_1_(*t*) after controlling for the effect of *x*_2_(*t*) localized in time and frequency. For a more detailed treatment of the partial wavelet coherence, we refer interested readers to Refs. [18, 19].

### Data

Here, we provide a detailed description of all analyzed series together with their source links. The characteristics of variables are described as of the time of the analysis, i.e. April 2014.

#### Bitcoin price index

The Bitcoin price index (BPI) is an index of the exchange rate between the US dollar (USD) and the Bitcoin (BTC). There are various criteria for specific exchanges to be included in BPI, which are currently (when the analysis was undertaken) met by three exchanges:Bitfinex, Bitstamp and BTC-e. Historically, Mt. Gox exchange was part of the index as well, but following its closure, the criteria ceased to be fulfilled. BPI is available on a 1-min basis, and it is formed as a simple average of the covered exchanges. The series are freely available at http://www.coindesk.com/price. Due to data availability, we analyze the relationships starting from 14 September 2011.

#### Blockchain

Blockchain (http://www.blockchain.info) freely provides very detailed series about Bitcoin markets. On a daily basis, the following time series used in our analysis are reported:

- Total bitcoins in circulation
- Number of transactions excluding exchange transactions
- Estimated output volume
- Trade volume vs. transaction volume ratio
- Hash rate
- Difficulty

The total number of bitcoins in circulation is given by a known algorithm and asymptotically until it reaches 21 million bitcoins. The creation of new bitcoins is driven and regulated by difficulty that mirrors the computational power of bitcoin miners (hash rate). Bitcoin miners certify ongoing transactions and the uniqueness of the bitcoins by solving computationally demanding tasks, and they obtain new (newly mined) bitcoins as a reward. Rewards and difficulties are given by a known formula.

The Bitcoin is used primarily for two purposes:purchases and exchange rate trading. Blockchain provides the total number of transactions and their volume excluding the exchange rate trading (exchange transactions). In addition, the ratio between volume of trade (primarily purchases) and exchange transactions is provided. Understandably, the over-the-counter (OTC) transactions are not covered.

#### Exchanges

Time series of exchange rates between BTC and various currencies are available at http://www.bitcoincharts.com. There, we obtain exchange volumes as a sum of four of the most important exchanges—Bitfinex, Bitstamp, BTC-e and Mt. Gox—which account for more than 90% of all USD exchange transactions on the Bitcoin markets. Although Mt. Gox is already in insolvency, we include it in the total exchange volume because it was the biggest exchange until 2013 and its exclusion would thus strongly bias the actual volumes. After its bankruptcy, the volumes converged to zero. For an examination of the relationship between the USD and Chinese Renminbi (CNY) Bitcoin markets, we use prices and volumes of the btcnCNY market, which is by far the biggest CNY exchange.

#### Search engines

We utilize data provided by Google Trends at http://trends.google.com and Wikipedia at http://stats.grok.se. For both, we are interested in the term “Bitcoin”. Google Trends standardly provides weekly data, whereas the Wikipedia series are daily. To obtain daily series for Google searches, one needs to download Google Trends data in three months blocks. The series are then chained and rescaled using the last overlapping month.

#### Financial Stress Index

The Financial Stress Index (FSI) is provided by the Federal Reserve Bank of Cleveland at https://www.clevelandfed.org/research/data/financial_stress_index/. The FSI can be separated into various components. However, we use the overall index to control for all types of financial stress.

#### Gold price

Gold prices for a troy ounce are obtained from https://www.gold.org/research, and we use prices in Swiss francs (CHF) due to its stability and lack of expansive monetary policy. However, the results remain largely the same regardless of the used currency.

According to Grinsted et al. [14], the series examined using the wavelet methodology should not be too far from a Gaussian distribution and primarily not multimodal. If the series are in fact multimodal, it is suggested that they be transformed to a uniform distribution and that quantiles of the original series, in turn, be analyzed. The inference based on the wavelet framework and the related Monte Carlo simulations based significance is then reliable. For this matter, we transform all of the original series accordingly, as most of them and particularly the Bitcoin price, are multimodal, and we thus interpret the results based on the quantile analysis.

## Results

We analyze drivers of the exchange rate between the Bitcoin (BTC) and the US dollar (USD) between 14.9.2011 and 28.2.2014. This specific exchange rate pair is selected because trading volumes on the USD markets form a strong majority, followed by a profound lag by the Chinese renminbi (CNY). The analyzed period is restricted due to the availability of a Bitcoin price index covering the most important USD exchanges. Note that an analysis of a specific exchange is not feasible because the most important historical market, Mt. Gox, filed for bankruptcy after serious problems with bitcoin withdrawals in 2014. For this reason, we use the CoinDesk Bitcoin price index (BPI), which is constructed as the average price of the most liquid exchanges. Please refer to the Methods section for further details about BPI.

**Fig 1:**
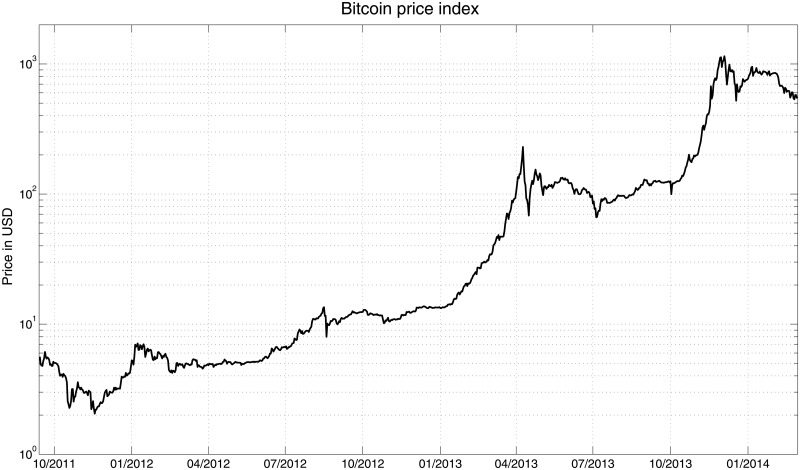
Bitcoin price index. Values of the index are shown in the USD (for the USD markets) and in the logarithmic scale.

Evolution of the price index is shown in Fig 1, in which we observe that the Bitcoin price is dominated by episodes of explosive bubbles followed by corrections, which never return to the starting value of the pre-bubble phase. The analyzed period starts with a value of approximately $5 per bitcoin and ends at approximately $600. Although the most recent dynamics of the Bitcoin price can be described as a slow decreasing trend, the potential profit of a buy-and-hold strategy of almost 12000% in less than 30 months remains appealing.

Compared with standard currencies such as the US dollar, the Euro, and the Japanese Yen, the Bitcoin shines due to the unprecedented data availability. It is completely unrealistic to know the total amount of US dollars in the worldwide economy on a daily basis. In a similar manner, it is also impossible to track the number of transactions that occur using the USD or other currencies. However, the Bitcoin provides this type of information on daily basis, publicly and freely. Such data availability allows for more precise statistical analysis. We examine Bitcoin prices considering various aspects that might influence the price or that are often discussed as drivers of the Bitcoin exchange rate. We start with the economic drivers, or potential fundamental influences, followed by transaction and technical drivers, influences on the interest in the Bitcoin, its possible safe haven status; finally, we focus on the effects of the Chinese Bitcoin market.

### Economic drivers

In economic theory, the price of a currency is standardly driven by its use in transactions, its supply and the price level. Either the time series for all of these variables are available or we are able to reconstruct them from other series; see the Methods section for more details.

**Fig 2:**
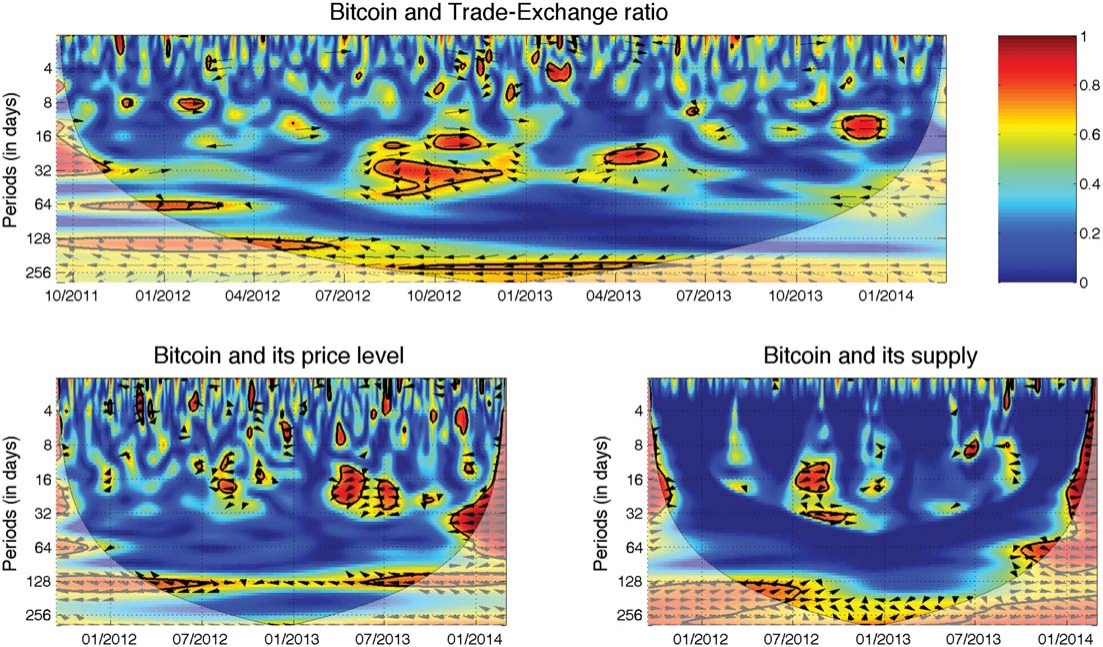
Fundamental drivers. Wavelet coherence is represented by a colored contour:the hotter the color is, the higher the local correlation in the time-frequency space (with time on the *x*-axis and scale on the *y*-axis). The matching of colors and correlation levels is represented by the scale on the right hand side of the upper graph. Regions with significant correlations tested against the red noise are contrasted by a thick black curve. The cone of influence separating the regions with reliable and less reliable estimates is represented by bright and pale colors, respectively. Phase (lag-lead) relationships are shown by the arrows—a positive correlation is represented by an arrow pointing to the right, a negative correlation by one to the left, leadership of the first variable is shown by a downwards pointing arrow and if it lags, the relationship is represented by an upward pointing arrow. The latter two relationships hold for the in-phase relationship (positive correlation); for the anti-phase (negative correlation), it holds vice versa. Henceforth, specifically for the fundamental drivers, Bitcoin price is negatively correlated to the Trade-Exchange ratio *(top)* over the long-term for the entire analyzed period, and there is no evident leader in the relationship. The Bitcoin price level is negatively correlated with the Bitcoin price in the long-term for the entire analyzed period as well *(bottom left)*, with no evident leader. For the relatively calm period between 05/2013 and 09/2013, the price level led the prices in the medium term. The supply of bitcoins is positively correlated with the price in the long-term *(bottom right)*, with no evident leader.

As a measure of the transactions use, i.e., demand for the currency, we use the ratio between trade and exchange transaction volume, which we abbreviate to Trade-Exchange ratio. The ratio thus shows what the ratio is between volumes on the currency exchange markets and in trade (e.g., purchases, services). Therefore, the lower the ratio is, the more frequently bitcoins are used for “real world” transactions. From the theory, the price of the currency should be positively correlated with its usage for real transactions because this increases the utility of holding the currency, and the usage should be leading the price. In Fig 2, we show the squared wavelet coherence between the Bitcoin price and the ratio. We thus see the evolution of the local correlation in time and across frequencies. The hotter the color is, the higher the correlation. Statistically significant correlations are highlighted by a thick black curve around the significant regions; significance is based on Monte Carlo simulations against the null hypothesis of the red noise, i.e., an autoregressive process of order one. The cone of influence separates the reliable (full colors) and less reliable (pale colors) regions. A phase difference, i.e., a lag or lead relationship, is represented by oriented arrows. Please refer to the Methods section for more detail. Specifically for the Trade-Exchange ratio, we observe a strong, but not statistically significant at the 5% level, relationship at high scales. The variables are in the anti-phase, so they are negatively correlated in the long term. However, there is no strong leader in the relationship. The slightly dominating frequency of the arrows pointing to the southwest hints that the ratio is a weak leader. On the shorter scales, most of the arrows point to the northeast, indicating that the variables are positively correlated and that the prices lead the Trade-Exchange ratio. Note that this relationship is visible primarily for the periods with extreme price increases for the BTC. In other words, the Bitcoin appreciates in the long run if it is used more for trade, i.e., non-exchange transactions, and the increasing price boosts the exchange transactions in the short run. The former is thus consistent with the theoretical expectations, and the latter shows that increasing prices—potential bubbles—boost demand for the currency at the exchanges. Therefore, the Bitcoin behaves according to the standard economic theory, specifically the quantity theory of money, in the long run but it is prone to bubbles and busts in the short run. The former finding might be seen as surprising given an unorthodox functioning of the Bitcoin, and the latter one is in hand with previous empirical studies [10, 11].

Price level is an important factor because of an expectation that goods and services should be available for the same, or at least similar, price everywhere and that misbalances are controlled for by the exchange rate. This is referred to as the law of one price in the standard economic theory. When the price level associated with one currency decreases with respect to the price level of another currency, the first currency should be appreciating and its exchange rate should thus be increasing. An expected causality goes from the price level to the exchange rate (price) of the Bitcoin. The price level in our case is constructed as the average price of a trade transaction for a given day. Fig 2 uncovers that the most stable interactions take place at high scales at approximately 128 days. The relationship is negative as expected, but the leader is not clear. There is also a significant region at lower scales at approximately one month between 04/2013 and 07/2013. The relationship is again negative as expected, but the leadership of the price level is more evident here. Most of the other significant correlations are outside the reliable region. Again, the Bitcoin behavior does not contradict the standard monetary economics in the long run.

The money supply works as a standard supply, so that its increase leads to a price decrease. A negative relationship is thus expected. Moreover, due to a known algorithm for bitcoin creation, only long-term horizons are expected to play a role. In Fig 2, we observe that there is a relationship between the Bitcoin price and its supply. However, most of the significant regions are outside of the reliable region. Moreover, the orientation of the phase arrows is unstable, so it is not possible to detect either a sign or a leader in the relationship. This difficulty might be due to the fact that both the current and the future money supply is known in advance, so that its dynamics can be easily included in the expectations of Bitcoin users and investors. The expectations of the future money supply is thus incorporated into present prices and relationship between the two is in turn negligible.

### Transaction drivers

**Fig 3:**
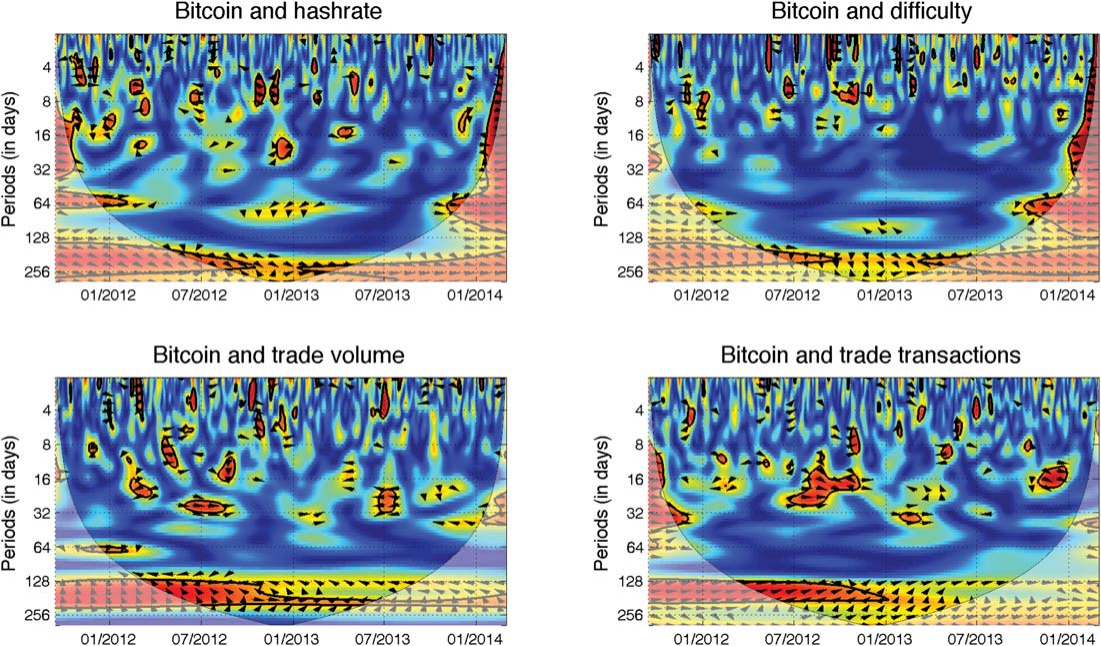
Currency mining and trade usage. The descriptions and interpretation of relationships hold from Fig 2. Both the hash rate *(top left)* and difficulty *(top right)* are positively correlated with the Bitcoin price in the long-term. The price leads both relationships as the phase arrow points to southeast in most cases, and the interconnection remains quite stable in time. The trade volume *(bottom left)* is again connected to the Bitcoin price primarily in the long-term. However, the relationship is not very stable over time. Until 10/2012, we observe a negative correlation between the two, and the price is the leader. The relationship then becomes less significant and the leader position is no longer evident. For the trade transactions *(bottom right)*, the relationship is positive in the long-term, and the transactions lead the Bitcoin price. However, the relationship becomes weaker over time, and it is not statistically significant from 01/2013.

The use of bitcoins in real transactions is tightly connected to fundamental aspects of its value. However, there are two possibly contradictory effects between the usage of bitcoins and their price, which might be caused by its speculative aspect. One effect stems from a standard expectation that the more frequently the coins are used, the higher their demand—and thus their price—will become. However, if the price is driven by speculation, volatility and uncertainty regarding the price, as well as the increasing USD value of transaction fees, can lead to a negative relationship. Trade volume and trade transactions are used as measures of usage. In Fig 3, we observe that for both variables, the significant relationships take place primarily at higher scales and occur primarily in 2012. The effect diminishes in 2013; and at lower scales, the significant regions are only short-lived and can be due to statistical fluctuations and noise. For the trade transactions, it is clear that the relationship is positive and that the transactions lead the price, i.e., the increasing usage of bitcoins in real transactions leads to an appreciation of the Bitcoin in the long run. However, the effect becomes weaker in time. For the trade volume, the relationship changes in time, and the phase arrows change their direction too often to offer us any strong conclusion. The transaction aspect of the Bitcoin value seems to be losing its weight in time.

### Technical drivers

Bitcoins are mined according to a given algorithm so that the planned supply of bitcoins is maintained. Miners, who mine new bitcoins as a reward for the certification of transactions in blocks, thus provide an inflow of new bitcoins into circulation. However, mining is contingent on solving a computationally demanding problem. Moreover, to keep the creation of new bitcoins in check and following the planned formula, the difficulty of solving the problem increases according to the computational power of the current miners. The difficulty is then provided by the minimal needed computational efficiency of miners, and it reflects the current computational power of the system measured in hashes. The hash rate then becomes another measure of system productivity, which is reflected in the system difficulty, which in turn is recalculated every 2016 blocks of 10 minutes, i.e., approximately two weeks. In this manner, the bitcoin supply remains balanced and the system is not flooded with bitcoins. Bitcoin mining is thus an investment opportunity in which computational power is exchanged for bitcoins. The mining itself is connected with the costs of the investment in hardware as well as electricity. Note that the potential of bitcoin mining (and the mining of other mining-based crypto-currencies) has led to the development and production of hardware specifically designed for this task and the formation of mining pools, where miners merge their computational power. The specialized equipment has led to the increasing costs of mining and a soaring mining hash rate and difficulty, which have gradually driven small miners away from the pools as mining became un-profitable for them.

There are again two opposing effects between the Bitcoin price and the mining difficulty as well as the hash rate. Mining can be seen as a type of investment in bitcoins. Rather than buying bitcoins directly, the investor invests in the hardware and obtains the coins indirectly through mining. This strategy leads to two possible effects. The increasing price of the Bitcoin can motivate market participants to start investing in hardware and start mining, which leads to an increased hash rate and, in effect, to a higher difficulty. Alternatively, the increasing hash rate and the difficulty connected with increasing cost demands for hardware and electricity drive more miners out of the mining pool. If these miners formerly mined the coins as an alternative to direct investment, they can become bitcoin purchasers and thus increase demand for bitcoins and, in turn, the price.

Fig 3 summarizes the wavelet coherence for both hash rate and difficulty. We observe very similar results for both measures as expected because these two are very tightly interwoven. Both measures of the mining difficulty are positively correlated with the price at high scales, i.e., in the long run, for almost the whole analyzed period. The relationship is clearer for the difficulty, which shows that Bitcoin price leads the difficulty, though the leadership becomes weaker over time. The effect of increasing prices attracting new miners thus appears to dominate the relationship. The weakening of the relationship over time can be attributed to the current stable or slowly decreasing price of bitcoins, which no longer offsets the cost of the computational power needed for successful mining. Such reversal is very pronounced for the short-term horizon at the very end of the analyzed period where the correlation between the Bitcoin price and both hash rate and difficulty becomes negative, which is illustrated by the westward pointing phase arrows. Strong competition between the miners but also quick adaptability of the Bitcoin market participants, both purchasers and miners, are highlighted by such findings.

### Interest

One of possible drivers of the Bitcoin price is its popularity. Simply put, increasing interest in the currency, connected with a simple way of actually investing in it, leads to increasing demand and thus increasing prices. To quantify the interest in the Bitcoin, we utilize Google and Wikipedia engines search queries for the word “Bitcoin”. It is obviously difficult to distinguish between various motives of internet users searching for information about the Bitcoin.

**Fig 4:**
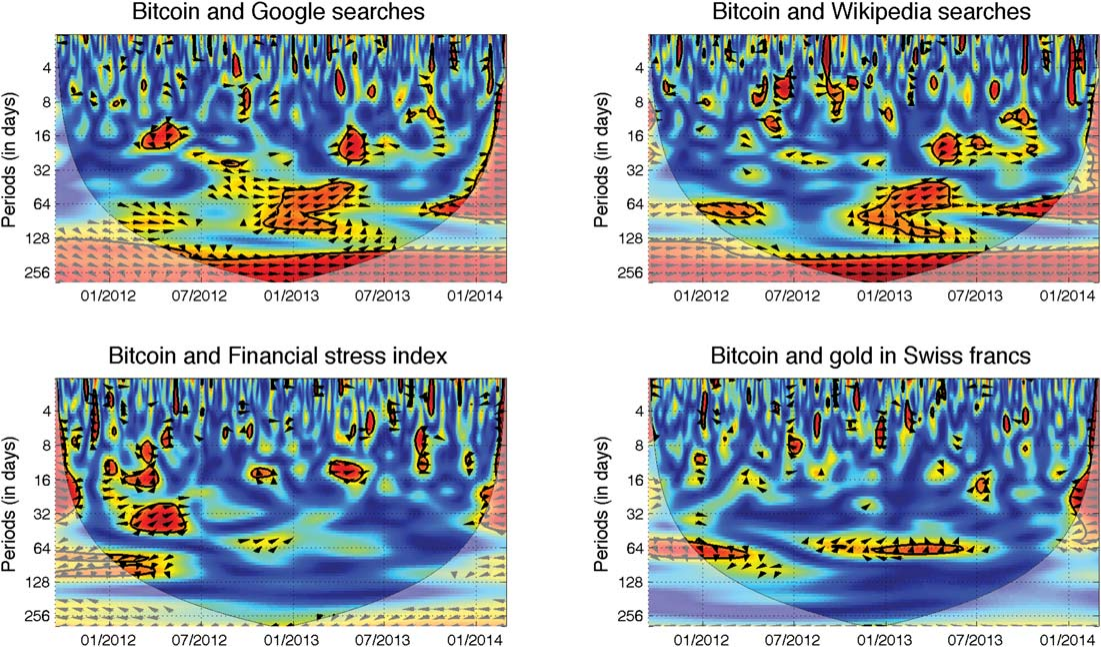
Search engines and safe haven value. The descriptions and interpretation of relationships hold from Fig 2. Searches on both engines *(top)* are positively correlated with the Bitcoin price in the long run. For both, we observe that the relationship somewhat changes over time. In the first third of the analyzed period, the relationship is led by the prices, whereas in the last third of the period, the search queries lead the prices. Unfortunately, the most interesting dynamics remain hidden in the cone of influence, and this result is thus not very reliable. Apart from the long run, there are several significant episodes at the lower scales with varying phase directions, hinting that the relationship between search queries and prices depends on the price behavior. Moving to the safe haven region, we find no strong and lasting relationship between the Bitcoin price and either the financial stress index *(bottom left)* or gold price *(bottom right)*. The significant regions at medium scales for gold are generally connected to the dynamics of the Swiss franc exchange rate.

In Fig 4, we show the wavelet coherence between the Bitcoin price and search engine queries. We observe that both search engines provide very similar information. The co-movement is the most dominant at high scales. However, we observe that the relationship changes over time. Up to the half of 2012, prices lead interest, and this relationship is more evident for the Google searches. The directionality of the relationship then becomes weaker, and starting from the beginning of 2013, it is hard to confidently discern the leader, though the searches tend to boost the prices. Nonetheless, the leadership is not very apparent. Apart from the long-term relationship, there are other interesting periods during which the interest in the coins and the prices are interconnected. The most visible of these periods takes place between 01/2013 and 04/2013 at medium scales between approximately 30 and 100 days. The prices are evidently led by interest in the Bitcoin during this period. Note that the first quarter of 2013 was connected to an exploding bubble during which the Bitcoin rocketed from $13 to above $200. Similar dynamics appear to be present also for the other bubble starting in 10/2013. Unfortunately, the entire development of this latter bubble is hidden in the cone of influence, and the findings are thus not statistically reliable. Addressing the 01/2013—04/2013 bubble, its deflation is also connected to the increased interest of internet users. The interest and prices are then negatively correlated, and the interest still leads the relationship. However, the correlations are found at lower scales than for the bubble formation. The interest in Bitcoin thus appears to have an asymmetric effect during the bubble formation and its bursting—during the bubble formation, interest boosts the prices further, and during the bursting, it pushes them lower. Moreover, the interest influence happens at different frequencies during the bubble formation and its bursting, so that the increased interest has a more rapid effect during the price contraction than during the bubble build-up. These results are in hand with Refs. [10, 11] who focus on the feedback effect between the Bitcoin and online attention in more detail.

### Safe haven

Though it might appear to be an amusing notion, the Bitcoin was also once labeled a safe haven investment. This label appeared during the Cypriot economic and financial crisis that occurred in the beginning of 2012. There were speculations that some of the funds from the local banks were transferred to Bitcoin accounts, thus ensuring their anonymity. Leaving these speculations aside, we quantitatively analyze the possibility of the Bitcoin being a safe haven. Specifically, we examine the relationship of Bitcoin prices with the Financial Stress Index (FSI) and the gold price in Swiss francs. The former is a general index of financial uncertainty. The latter combination of gold and Swiss franc are chosen because gold is usually considered to provide the long-term storage of value and the Swiss franc is considered to be a very stable currency, being frequently labeled as a safe haven itself. If the Bitcoin were truly a safe haven, it would be positively correlated with both utilized series, assuming that both FSI and gold price are good proxies of a safe haven.

Fig 4 summarizes the results. For the FSI, we observe that there is actually only one period of time that shows an interesting interconnection between the index and the Bitcoin price. This period is exactly that of the Cypriot crisis, and most of the co-movements are observed at scales around 30 days. Increasing FSI leads the Bitcoin price up. However, apart from the Cypriot crisis, there are no longer-term time intervals during which the correlations are both statistically significant and reliable (in the sense of the cone of influence). Turning now to the gold price, there appears to be practically no relationship apart from two significant islands at scales of approximately 60 days. However, these islands are most probably connected to the dynamics of gold itself because the first significant period coincides with a rapid increase in the gold price culminating around September 2011 (a large proportion of the significant region is outside of the reliable part of the coherence) and the second collides with the stable decline of gold prices. It thus appears that the Bitcoin is not connected to the dynamics of gold, but even more, it is not obvious whether gold still remains the safe haven that it once was. Either way, we find no sign that the Bitcoin is a safe haven, which is in fact expected considering the present behavior and (in)stability of prices.

### Influence of China

There are claims that events happening on the Chinese Bitcoin market have a significant impact on the USD markets. Some of the extreme drops as well as price increases in the Bitcoin exchange rate do coincide with dramatic events in China and Chinese regulation of the Bitcoin. Probably the most notable example are the developments around Baidu, which is an important player in Chinese online shopping. The announcement that Baidu was accepting bitcoins in mid-October 2013 started a surge in its value that was, however, cut back by Chinese regulation banning the use of bitcoins for electronic purchases in early-December 2013. The Chinese market is thus believed to be an important player in digital currencies and especially in the Bitcoin. To examine the relationship between the Chinese renminbi (CNY) and the US dollar markets, we look at their prices and exchange volumes.

**Fig 5:**
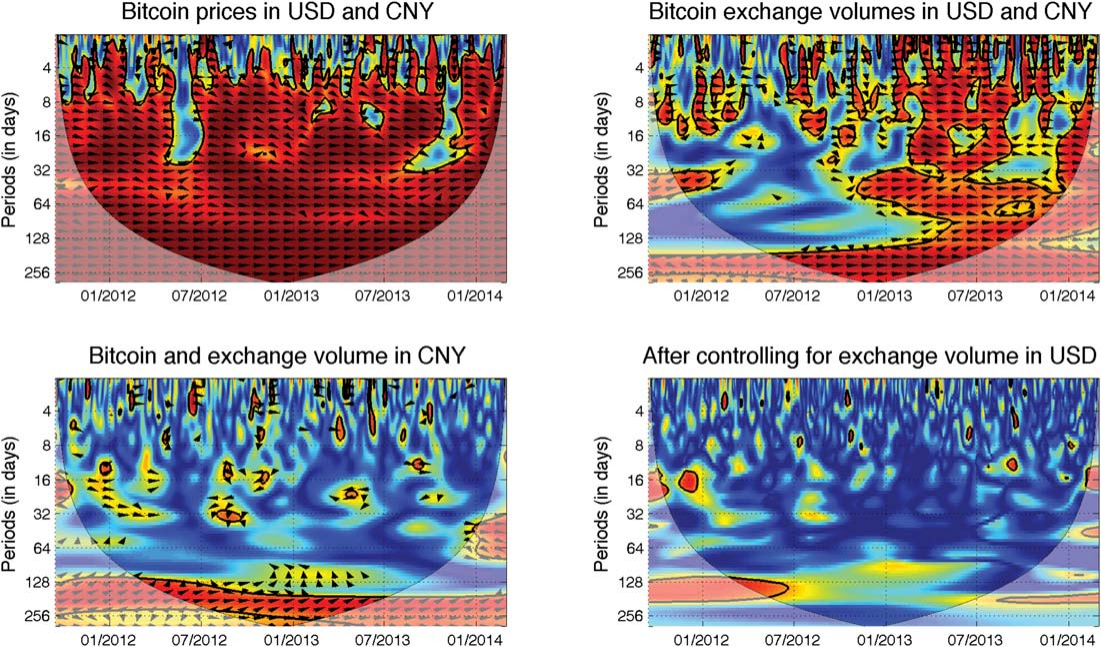
Influence of the Chinese market. The description and interpretation of relationships hold from Fig 2. Bitcoin prices in USD and CNY *(top left)* move together at almost all scales and during the entire examined period. There is no evident leader in the relationship, though the USD market appears to slightly lead the CNY at lower scales. However, at the lowest scales (the highest frequencies), the correlations vanish. For the volumes *(top right)*, the two markets are strongly positively correlated at high scales. However, for the lower scales, the correlations are significant only from the beginning of 2013 onwards. There is again no dominant leader in the relationship. The CNY exchange volume then leads the USD prices in the long run *(bottom left)*. However, when we control for the effect of the USD exchange volume *(top right)*, we observe that the correlations vanish.

Fig 5 includes all of the interesting results. The prices in both markets are tightly connected, and we observe strong positive correlations at practically all scales and during the entire examined period. From the phase arrows, we can barely find a leader in the relationship. More interesting dynamics are found for the exchange volumes. Here, we find that the volumes are strongly positively correlated as well, but only from the beginning of 2013 onwards. Before that period, the interconnections are visible only at the highest scales, and most of the dynamics fall outside the reliable region. Note that the trading volumes on the CNY market were quite low during 2012. In the significant section, we again find that the relationship is strong, and it is not easy to find an evident leader. Nonetheless, the period between 10/2013 and 12/2013 is again connected to the decoupling of markets similar to the connection for the prices. From these results, we can conclude that both markets tend to move together very tightly in terms of both price and volume.

One might believe that if the Chinese market is an important driver of the BTC exchange rate with the USD, an increased exchange volume in China might increase demand in all markets, so that the Chinese volume and the USA price would be connected. This connection is even more stressed by the fact that the shorting (selling now and buying later) of bitcoins is still limited. In Fig 5, we show that this connection does indeed exist, and the relationship is again present at high scales. Because most of the phase arrows point toward the northeast region, the Chinese volume leads the USD prices. However, as discussed above, the USD and CNY exchange volumes are strongly correlated, and at high scales, this is true for the entire analyzed period. Therefore, a relationship between CNY volume and USD price might be spuriously found due to this type of correlation. To control for this effect, we utilize partial wavelet coherence, which filters this effect away. In the last chart of Fig 5, we show that after controlling for the exchange volume of the USD market, practically no interconnection between the CNY volume and the USD price remains. Overall, we find no causal relationship between the CNY and the USD markets in the analyzed dataset. Nevertheless, this does not discard possible causal relationship at even lower scales, i.e., in the high-frequency domain. This suggests that the USD and CNY Bitcoin markets react to the relevant news quickly so that there is no lead-lag relationship at scales of one day or higher. Such property can be likely attributed to the algorithmic trading which efficiently seeks arbitrage opportunities between different Bitcoin exchanges.

## Discussion

Bitcoin price dynamics have been a controversial topic since the crypto-currency increased in popularity and became known to a wider audience. We have addressed the issue of Bitcoin price formation and development from a wider perspective, and we have investigated the most frequently claimed drivers of the prices. There are several interesting findings. First, although the Bitcoin is usually considered a purely speculative asset, we find that standard fundamental factors—usage in trade, money supply and price level—play a role in Bitcoin price over the long term. These findings are well in hand with standard economic theory, and specifically monetary economics and the quantity theory of money. Second, from a technical standpoint, the increasing price of the Bitcoin motivates users to become miners. However, the effect is found to be vanishing over time time, as specialized mining hardware components have driven the hash rates and difficulty too high. Nonetheless, this is a standard market reaction to an obvious profit opportunity. A reversal is identified at the end of the analyzed period. Third, the prices of bitcoins are driven by investors’ interest in the crypto-currency. The relationship is most evident in the long run, but during episodes of explosive prices, this interest drives prices further up, and during rapid declines, it pushes them further down. This is well in hand with previous research on the topic [10, 11]. Fourth, the Bitcoin does not appear to be a safe haven investment. Finally, fifth, although the USD and CNY markets are tightly connected, we find no clear evidence that the Chinese market influences the USD market. We speculate that such behavior is due to the analyzed data structure and its frequency, and trading algorithms which efficiently capitalize on potential arbitrage opportunities between different Bitcoin exchanges. Overall, the Bitcoin forms a unique asset possessing properties of both a standard financial asset and a speculative one.
